# Patient Uptake, Experience, and Satisfaction Using Web-Based and Face-to-Face Hearing Health Services: Process Evaluation Study

**DOI:** 10.2196/15875

**Published:** 2020-03-20

**Authors:** Husmita Ratanjee-Vanmali, De Wet Swanepoel, Ariane Laplante-Lévesque

**Affiliations:** 1 Department of Speech-Language Pathology & Audiology University of Pretoria Pretoria South Africa; 2 Ear Sciences Centre The University of Western Australia Nedlands Australia; 3 Ear Science Institute Australia Subiaco, Western Australia Australia; 4 Oticon Medical A/S Copenhagen Denmark; 5 Department of Behavioural Sciences and Learning Linköping University Linköping Sweden

**Keywords:** audiology, hearing loss, internet-based intervention, patient outcome assessment, patient satisfaction, telemedicine, text messaging, eHealth, mHealth, social media, patient-centered care

## Abstract

**Background:**

Globally, access to hearing health care is a growing concern with 900 million people estimated to suffer from disabling hearing loss by 2050. Hearing loss is one of the most common chronic health conditions, yet access to hearing health care is limited. Incorporating Web-based (voice calling, messaging, or emailing) service delivery into current treatment pathways could improve access and allow for better scalability of services. Current electronic health studies in audiology have focused on technical feasibility, sensitivity, and specificity of diagnostic hearing testing and not on patient satisfaction, experiences, and sustainable models along the entire patient journey.

**Objective:**

This study aimed to investigate a hybrid (Web-based and face-to-face) hearing health service in terms of uptake, experience, and satisfaction in adult patients with hearing loss.

**Methods:**

A nonprofit hearing research clinic using online and face-to-face services was implemented in Durban, South Africa, using online recruitment from the clinic’s Facebook page and Google AdWords, which directed persons to an online Web-based hearing screening test. Web-based and face-to-face care pathways included assessment, treatment, and rehabilitation. To evaluate the service, an online survey comprising (1) a validated satisfaction measurement tool (Short Assessment of Patient Satisfaction), (2) a process evaluation of all the 5 steps completed, and (3) personal preferences of communication methods used vs methods preferred was conducted, which was sent to 46 patients who used clinic services.

**Results:**

Of the patients invited, 67% (31/46) completed the survey with mean age 66 years, (SD 16). Almost all patients, 92% (30/31) reported that the online screening test assisted them in seeking hearing health care. Approximately 60% (18/31) of the patients accessed the online hearing screening test from an Android device. Patients stayed in contact with the audiologist mostly through WhatsApp instant messaging (27/31, 87%), and most patients (25/31, 81%) preferred to use this method of communication. The patients continuing with hearing health care were significantly older and had significantly poorer speech recognition abilities compared with the patients who discontinued seeking hearing health care. A statistically significant positive result (*P*=.007) was found between age and the number of appointments per patient. Around 61% (19/31) of patients previously completed diagnostic testing at other practices, with 95% (18/19) rating the services at the hybrid clinic as better. The net promoter score was 87, indicating that patients were highly likely to recommend the hybrid clinic to friends and family.

**Conclusions:**

This study applied Web-based and face-to-face components into a hybrid clinic and measured an overall positive experience with high patient satisfaction through a process evaluation. The findings support the potential of a hybrid clinic with synchronous and asynchronous modes of communication to be a scalable hearing health care model, addressing the needs of adults with hearing loss globally.

## Introduction

### Background

Globally, access to hearing health care (HHC) is a significant challenge affecting 466 million people, and this number is expected to rise to 900 million people by 2050, who are estimated to have disabling hearing loss [1]. The limited access to HHC results in most affected persons to live with untreated hearing loss, which has far-reaching consequences for individuals and the society at large [2]. Untreated hearing loss affects health, independence, well-being, and employment opportunities and is associated with social isolation, depression, and an increased risk of dementia [3-8]. Alongside recent estimates of a global cost of US $750 billion to hearing loss [1], this chronic condition is now recognized as a significant public health concern [9,10].

### Hearing Health Care Models

Traditional HHC service delivery models focus on face-to-face, clinic-based testing, hearing aid or device fittings, counseling, and rehabilitation requiring several patient visits. HHC can be made more accessible through scalable models of care that capitalize on global trends in connectivity and technology [11]. For example, by the end of 2018 there were 5.1 billion mobile subscribers, which represents 67% of the global population, and 3.6 billion mobile device internet users, which accounts for 47% of the global population [12].

The use of these telecommunication and information technologies in medicine is called telemedicine or telehealth; in the field of ear and hearing health, the terms tele-otology and tele-audiology are also used [13]. Owing to the lack of consistency and confusion, many professionals have adapted their own term, ie, electronic health (eHealth), telehealth, tele-audiology, and now eAudiology are all terms that are often used interchangeably to describe the dissemination of health or hearing health services using the internet [14]. Although tele-practice was initially intended for services to be delivered to individuals at a distance, where patients could not interact with health professionals or the patient and the health professional were at two different locations, a newer approach is to provide HHC to the patient who may be close in distance to the health professional but chooses tele-practice as a service delivery option out of convenience [14]. Telehealth relies on access to the internet, and while some communities may have limited access, connectivity is rapidly expanding [12,15].

### TeleHealth in Hearing Health Care

There is a growing body of evidence on the use of telehealth in HHC, including screening [16,17] diagnostic assessment [18,19], hearing aid fitting [20,21], and rehabilitation [22,23]. Studies to date have tested the use of tele-audiology at specific points along the patient journey and have mostly been proof-of-concept studies [13,15,24,25] that have not translated into sustainable telehealth practices [24]. There is a significant need to not only evaluate service delivery models that incorporate telehealth approaches along the patient journey in terms of effectiveness and efficiency but also to establish patient acceptance and satisfaction [13]. Measuring patient outcomes is important, as positive outcomes indicate improvements on patient satisfaction, adherence, and health status [26]. This therefore highlights the need for measuring patient satisfaction.

A dearth of evidence on patient satisfaction when using telehealth HHC services is apparent [13], as only a few studies report on patient satisfaction with tele-audiology. In one study, patients who had their hearing aids fitted remotely were followed up upon, and a high level of patient satisfaction was noted [21]. In another study, there was no difference in terms of the hearing aid benefit between in-person and tele-audiology hearing aid services [22]. In these 2 studies, patient satisfaction with tele-audiology was measured only once, and the measurement was limited to treatment outcomes, rather than an indication of the process of receiving HHC services through a different service delivery medium.

Offering hearing services completely online along the entire patient journey is challenging. Online components were selected based on validated and evidence-based tools, which would not compromise the quality of patient care (eg, online hearing screening, communication by phone and WhatsApp, and online rehabilitation). These components (eg, video-otoscopy, audiological diagnostic evaluation, and real-ear measurements) were included in face-to-face appointments as online alternatives were not yet available at the conception of this study. The model is described further in the following section and in the study by Ratanjee-Vanmali et al [27].

In a previous study, we reported on the behaviors of participants who failed the online hearing screening test. Approximately 25% (13/51) of participants proceed from motivational engagement to diagnostic testing and the remainder 75% (38/51) do not transition for the following reasons: unanswered phone call, 45% (17/38), further investigation (curious about the online hearing screening test or owns hearing aids but wants a confirmation of hearing loss, 29% (11/38), incorrect contact details, 8%(3/38), doctor did not advocate for further treatment, 8%(3/38), limited finances, 8% (3/38), and beyond the test geolocation, 3% (1/38) [27]. Therefore, this highlights the need to understand patient experience, satisfaction, and engagement in seeking HHC through such a hybrid model and which components encourage them to continue to seek HHC.

### Objective of Study

This study aimed to describe a process evaluation of HHC through a hybrid clinic combining online and face-to-face services [27], with a focus on patient uptake, experience, and satisfaction.

## Methods

### Data Collection Procedure

The institutional review board approved the research (GW20170409HS).

#### Hybrid Service Delivery Model

This research project established a nonprofit hearing research clinic [28] in Durban, South Africa. The clinic relied on online patient recruitment, offering a free online hearing screening. Online recruitment using Facebook and Google was used to target adults aged ≥40 years within the target geolocation from the clinic’s social media account. Although the typical age for first-time hearing aid users is 74 years [29], the motivation for advertising to a younger audience was to reach the children of the parents aged 65 to 75 years who would resonate with the advertisements and share or encourage their family members to complete the online screening test. Advertisements (images and videos), articles, and blogs were created and used on the clinic’s Facebook page regarding the importance of HHC and knowing one’s hearing status or ability, and Google AdWords related to hearing test, audiologist, and tinnitus were used.

**Figure 1:**
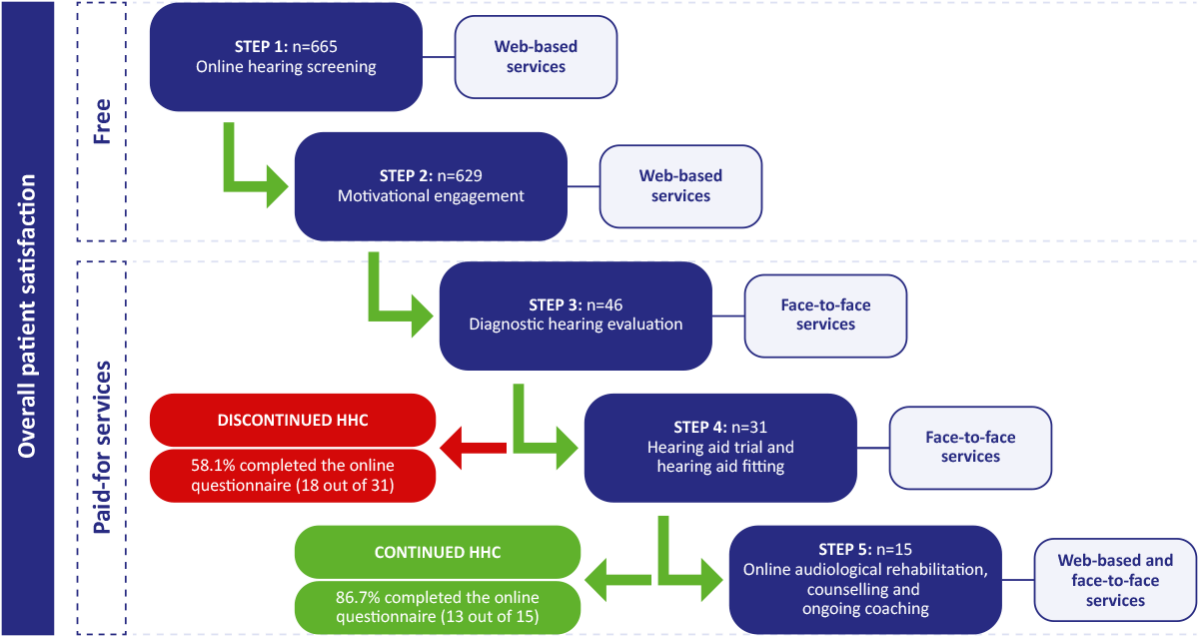
Five steps in a hybrid (Web-based and face-to-face) hearing health care (HHC) service-delivery model.

Upon completion, patients could opt to provide their contact details to be contacted by the clinic. If patients contacted the clinic without taking the online hearing screening test, they received a link encouraging them to complete the online test. At the beginning of every face-to-face appointment, the clinic audiologist verified the completion of the online hearing screening test. Asynchronous and synchronous online communication, as well as face-to-face communication supporting screening, diagnostics, hearing aid fitting, rehabilitation, and continuous monitoring and coaching, were offered. In total, 5 steps were included in the patient journey (Figure 1). The first 2 steps in the model (ie, Web-based hearing screening and motivational engagement, see the following section) were free. Participants paid for the 3 final steps, with some of the participants having access to reimbursement through their health insurance.

### Steps in the Hybrid Hearing Health Care Delivery Model

#### Step 1: Online Hearing Screening—Web-Based

The online hearing screening test is an adaptive triple digit–in–noise test developed and validated for South African English that determines a speech reception threshold [30,31]. The online hearing screening test that comprised 23 user entries was provided as a software-enabled Web widget [32] hosted on the clinic’s website.

When beginning the online hearing screening test, each participant was required to provide their date of birth. For each participant completing the online hearing screening test, the signal-to-noise ratio (SNR) where 50% of digits are recognized correctly, was recorded. The geolocation was also provided, which helped verify whether participants were within the geolocation of the test, ie, the greater Durban area. The pass or fail threshold of the online hearing screening test was based on optimal sensitivity and specificity to a 4-frequency pure tone average at 0.5, 1, 2, and 4 kHz ≤25 dB HL in the better ear.

On completion of the screening test, individuals were informed of their result in terms of pass or fail. Individuals could share their contact details if they wanted the clinic audiologist to contact them. The online hearing screening test results were stored in mHealth Studio Cloud; even if individuals did not share their contact details, the result was stored with an accurate geolocation which ensured that only data from the target location was used in the analysis [32]. Only the clinic audiologist had access to the password-protected mHealth Studio Cloud [32].

#### Step 2: Motivational Engagement—Web-Based

This step consisted of a phone call or WhatsApp message thread where the clinic audiologist assessed the readiness to book a face-to-face diagnostic hearing evaluation and provided motivational engagement.

Individuals who shared their contact details received an email with the clinic audiologist’s contact details, motivational engagement questions, and suitable times and dates for a phone call.

Readiness measurement and motivational engagement consisted of 2 validated tools: the line and staging algorithm that were used with the participant over the phone. The line is a single-item measure to assess readiness for hearing help-seeking in one question: “How important is it for you to improve your hearing right now?” Responses are recorded on a Likert scale from 0 to 10, where 0 indicates not at all and 10 indicates very much [33,34]. The staging algorithm is also a single-item question assessing the stages of change with 4 possible answers, each corresponding with a stage of change: (1) I do not think I have a hearing problem, and therefore nothing should be done about it (precontemplation); (2) I think I have a hearing problem. However, I am not yet ready to take any action to solve the problem, but I might do so in the future (contemplation); (3) I know I have a hearing problem, and I intend to take action to solve it soon (preparation); and (4) I know I have a hearing problem, and I am here to take action to solve it now (action) [35]. When participants scored above 5 on the Likert rating scale of 0 to 10 [33,34] and scored 3 or 4 in the staging algorithm [35], a face-to-face visit for the comprehensive hearing evaluation was scheduled. Higher ratings indicate greater readiness to take action.

#### Step 3: Diagnostic Hearing Evaluation—Face-to-Face

This step consisted of a face-to-face appointment where the clinic audiologist completed a battery of assessments including an in-depth case history, video-otoscopy, acoustic reflexes, pure tone audiometry (air and bone conduction), and speech audiometry. If no red flag (eg, sudden onset of hearing loss, middle ear pathology, and asymmetrical hearing loss, sudden onset of tinnitus, aural fullness, and vertigo) suggesting a medical referral was raised, a hearing aid trial was recommended.

#### Step 4: Hearing Aid Trial and Fitting—Face-to-Face

A successful hearing aid trial entailed that the patient acquired hearing aids fit according to their personalized gain setting, signal processing, noise management system, automatic systems, style, and color. During the hearing aid trial, a receiver in the ear with domes chosen to meet acoustic requirements was fit to meet the patient’s audiological profile. Patients were then offered a choice to opt for the style of hearing aids they preferred once counselled on the acoustic performance, physical characteristics of the available hearing aids, and personal needs (from in-the-ear custom options to behind-the-ear hearing aids). Trial hearing aids were then fit and customized to the audiometric profile of the patient using real-ear measurements to take individual ear canal properties into account.

#### Step 5: Audiological Rehabilitation, Counselling, and Ongoing Coaching—Web-Based and Face-to-Face

All patients who acquired hearing aids were offered an online audiological rehabilitation program [36] and the clinic audiologist coached them routinely.

The online audiological rehabilitation program consisted of 5 modules (becoming a successful hearing aid user; understanding my own hearing loss; handling my hearing aids; managing difficult communication situations; and communicating my own hearing loss) that are a combination of videos, tasks, testimonials, and reading assignments. The completion of a module would unlock the next module. The 5 modules were completed all at once or weekly as per the patient’s availability. Through prerecorded videos, a coach guided the participants through the different modules and components. More information regarding the hybrid clinic has been reported elsewhere [27].

### Materials

#### Online Questionnaire

An online questionnaire was used to determine the experience and satisfaction of patients seeking and receiving HHC using the hybrid clinic, incorporating online and face-to-face services. The online questionnaire was hosted and administered by Qualtrics (Provo, Utah) [37]. The responses were password protected and only accessible to the clinic audiologist. This closed survey was only administered to patients who provided consent to partake in the study. Participation was voluntary; no incentives were offered to encourage completion of the questionnaire.

The questionnaire (Multimedia Appendix 1) consisted of 3 sections totaling 41 questions for the group that discontinued HHC (exited at step 3) and 43 questions for the group that continued with HHC (exited at step 5). The 3 sections consisted of (1) a validated satisfaction measurement tool (Short Assessment of Patient Satisfaction [SAPS] [38]; (2) a process evaluation of all the 5 steps (online hearing screening, motivational engagement, diagnostic hearing evaluation, hearing aid trial and fitting, and online rehabilitation together with counseling and ongoing coaching) completed as seen in Figure 1; and (3) personal preferences of communication methods used versus methods preferred and HHC experiences compared with previous care, which were sent to 46 patients who used clinic services. Reporting of the questionnaire was separated into 2 overall sections: (1) evaluation of the steps and (2) patient experiences and satisfaction with the hybrid service delivery model.

The online questionnaire included a process evaluation, recorded on a 5-point Likert scale, which evaluated all the 5 steps (Figure 1). The method (Multimedia Appendix 2) was inspired by Linnan and Steckler [39]. They propose how to design and implement a process evaluation by creating the inventory of process objectives based on theory; reaching a consensus of the questions to be answered by the stakeholders of the project; identifying and creating the measurement tools; designing, implementing, and administering quality control; collecting, managing, and cleaning data; analyzing data; reporting findings; and refining interventions, measurements, and the analysis tool [39]. The process evaluation questionnaire was developed to include all aspects of the hybrid service delivery model which included and excluded a clinician’s involvement, where no systematic differences were found in the ratings. Closed and open-ended questions on patient experiences and preferences related to the hybrid clinic services compared with a traditional model were surveyed along with communication methods used and those preferred. Answers to open-ended questions were analyzed using an inductive thematic analysis that was conducted by the first author and then reviewed by an independent researcher [40].

#### Overall Satisfaction

SAPS [38] assesses overall patient satisfaction with 7 items targeting treatment, explanation of treatment results, clinician care, participation in medical decision making, respect by the clinician, time with the clinician, and satisfaction with hospital or clinic care. The questionnaire has been validated in clinical settings and has good internal and test-retest reliability [38]. For this study, the questionnaire was tailored to audiology by replacing the term doctor or other health professional to audiologist [41]. The minimum score is 0, and the maximum score is 28, where higher scores indicate greater satisfaction. Typical total SAPS scores from other research reported mean scores of 22 (SD 5) and 8 (SD 4) [38,42].

The net promoter score (NPS) is a single question about willingness to recommend a product or service that companies commonly use [43]: “On a scale from 0-10, how likely are you to recommend this clinic (Hearing Research Clinic NPC) to your friends and family?” A follow-up question asking respondents to explain the rating followed. The NPS is calculated by classifying the respondents into promoters (9-10), passives (7-8), and detractors (≤6). The NPS is obtained by subtracting the percentage of detractors from the percentage of promoters [43].

### Procedures

The online questionnaire was sent via email to 46 patients who completed the diagnostic hearing evaluation (steps 1-3). Data for each patient gathered from their files were linked by their email address and then hidden to ensure anonymity and were issued patient numbers during data analysis. Data were collected over 3 months (December 2018-February 2019); patients had sought help from the clinic during a period of 19 months (June 2017-January 2019). All patients who completed the survey were in contact with the clinic within 6 months of completing the online questionnaire.

The initial email invitation was sent on December 4, 2018, and a WhatsApp message was sent prompting patients to check their email mailboxes for the questionnaire. Up to seven reminder messages (email and WhatsApp) were sent to nonresponses over 12 weeks.

Patient data were stored in two locations: (1) a cloud-based system for appointment times and notes and (2) a server-based system for diagnostic results. Both systems were password protected and only accessible by the clinic audiologist.

### Participants

Purposive sampling was used to collect patients’ experiences and satisfaction of the hybrid clinic services. Patients who failed the online hearing screening provided consent to be contacted by the clinic audiologist before submitting their details. Written consent to partake in the study was provided during the face-to-face diagnostic hearing evaluation (step 3).

### Statistical Analysis

Data were analyzed using SPSS Inc, version 25 (IBM Corp, Chicago, Illinois) [44]. Statistical significance was set at *P*<.05. The Shapiro-Wilk test (nonparametric test) was used to test normality, which confirmed that the data were not normally distributed. Cronbach alpha was used to test the internal validity of the entire process evaluation questionnaire.

## Results

### Characteristics of Online Seekers of Hearing Health Care

The reporting of questionnaire results is in accordance, as far as possible, with the Checklist for Reporting Results of Internet E-Surveys [45].

A total of 665 participants completed the online hearing screening test and submitted their details for further HHC services during this evaluation period. A total of 629 participants were contacted by telephone or WhatsApp for motivational engagement; a few were unreachable owing to incorrect details submitted. Out of the 629 participants contacted, 46 (7%) became patients of the clinic and sought HHC services (Figure 1). Of the 46 patients invited, 31 (67%) completed the online survey and were aged between 35 and 101 years (mean 66, SD 16), the majority, 58% (n=18) being men. On average, patients had experienced hearing difficulties for 13 years (SD 15) and presented with an average speech reception threshold of −3.0 dB SNR (SD 8). The online questionnaire was internally consistent and reliable; Cronbach alpha values were between 0.70 and 0.77, where a value above 0.70 was considered acceptable [46]. No significant differences were found on the Mann-Whitney U test between the responder (n=31) and nonresponder (n=15) groups in terms of age, gender, SNR, the line, staging algorithm, years aware of hearing loss, and devices used to complete the online hearing screening test.

### Process Evaluation of 5 Steps in Hybrid Hearing Health Care Delivery Model

#### Step 1: Online Hearing Screening—Web-Based

Patients (N=31) accessed the online hearing screening from Android (18/31, 58%), iOS (9/31, 29%), and Windows PC (4/31, 13%) devices. The majority of patients agreed or strongly agreed that the online hearing screening was simple to complete (24/25, 96%), was quick and informative (23/26, 88%), was easy to use (23/26, 89%) and assisted them to continue HHC (24/26, 92%; Table 1).

**Table 1:** Patient evaluation of the online hearing screening test.

Questions related to the online hearing screening test	Strongly disagree, n (%)	Disagree, n (%)	Neutral, n (%)	Agree, n (%)	Strongly agree, n (%)
Taking the online test was simple (N=25)	0 (0)	1 (4)	0 (0)	14 (56)	10 (40)
Taking the online test was quick (N=26)	0 (0)	0 (0)	3 (12)	17 (65)	6 (23)
Taking the online test was informative (N=26)	0 (0)	0 (0)	3 (12)	17 (65)	6 (23)
I found this online test easy to use (N=26)	0 (0)	1 (4)	2 (8)	15 (58)	8 (31)
I thought the online test was fast (N=26)	0 (0)	5 (19)	7 (27)	9 (35)	5 (19)
The test result seemed reliable (N=26)	0 (0)	1 (4)	2 (8)	15 (58)	8 (31)
Online test has helped me to take the next steps to improve my hearing (N=26)	0 (0)	0 (0)	2 (8)	10 (39)	14 (54)

#### Step 2: Motivational Engagement—Web-Based

Patients agreed and strongly agreed that the mode of communication was easy (26/26, 100%), quick (27/27, 100%), provided useful (26/26, 100%) and relevant (25/26, 96%) information, assisted in taking the next step (25/26, 96%), and assisted in booking the diagnostic hearing evaluation (27/28, 96%); Table 2).

**Table 2:** Patient evaluation of motivational engagement using a voice call/messaging (WhatsApp).

Questions related to a voice call/messaging (WhatsApp)	Strongly disagree, n (%)	Disagree, n (%)	Neutral, n (%)	Agree, n (%)	Strongly agree, n (%)
The phone call/WhatsApp message was informative (N=26)	0 (0)	0 (0)	0 (0.)	15 (58)	11 (42)
The phone call/WhatsApp message was an easy way for me to communicate with the audiologist/clinic (N=26)	0 (0)	0 (0)	0 (0)	15 (58)	11 (42)
The phone call/WhatsApp message helped me in taking the next step (N=26)	0 (0)	0 (0)	1 (4)	11 (42)	14 (54)
The phone call/WhatsApp message provided me with relevant information regarding my hearing (N=26)	0 (0)	0 (0)	1 (4)	14 (54)	11 (42)
The phone call/WhatsApp message helped me to take the next step and book my hearing evaluation consultation (N=28)	0 (0)	0 (0)	1 (4)	12 (43)	15 (54)
The phone call/WhatsApp message was a quick way for me to communicate with the audiologist/clinic (N=27)	0 (0)	0 (0)	0 (0)	14 (52)	13 (48)

Patients communicated with the clinic using WhatsApp messaging (27/31, 87%), emails (25/31, 81%), voice calls (24/31, 77%), text messages (4/31, 13%), and Facebook Messenger (2/31, 7%). The majority of patients preferred the following methods of communication with the clinic audiologist: WhatsApp messaging (25/31, 81%), email (20/31, 65%), or voice calls (19/31, 61%).

#### Step 3: Diagnostic Assessment—Face-to-Face

Patients attending face-to-face diagnostic appointments agreed and strongly agreed that the test was comprehensive (31/31, 100%), provided the information needed (31/31, 100%), was easy to complete (31/31, 100%), and was trustworthy (31/31, 100%) with sufficient time spent taking it (31/31, 100%; Table 3).

**Table 3:** Patient evaluation of the diagnostic hearing evaluation.

Questions related to the diagnostic hearing evaluation	Strongly disagree, n (%)	Disagree, n (%)	Neutral, n (%)	Agree, n (%)	Strongly agree, n (%)
The diagnostic hearing test was comprehensive (N=31)	0 (0)	0 (0)	0 (0)	13 (42)	18 (58)
The audiological consultation provided me with the information I needed (N=31)	0 (0)	0 (0)	0 (0)	13 (42)	18 (58)
The diagnostic hearing test was an easy test to complete with the guidance from the audiologist (n=30)	0 (0)	0 (0)	0 (0)	9 (30)	21 (70)
It was beneficial to have a hearing aid trial option available after my diagnostic hearing test (in the first consultation; N=28)	0 (0)	0 (0)	1 (4)	6 (21)	21 (75)
It was easy to use the hearing aid during the trial period offered to me (N=28)	0 (0)	0 (0)	2 (7)	8 (29)	18 (64)
I trust the results from my diagnostic hearing test (N=30)	0 (0)	0 (0)	0 (0)	11 (37)	19 (63)
The time spent on my diagnostic hearing test was adequate (N=31)	0 (0)	0 (0)	0 (0)	13 (42)	18 (58)

More than half of the patients (19/31, 61%) had previously completed a diagnostic hearing evaluation (step 3) at other practices. In comparison with previous experiences, one person rated the hybrid clinic as the same while the other 18 patients rated their experiences as better.

From the open-ended responses, two main themes emerged for the differences between prior experiences and the hybrid clinic: clinician engagement and technology. Clinician engagement included aspects of personal attention, patience, dedication, thorough explanations, professional behavior, exceeding expectations, friendliness, and trust. Technology included aspects of the latest technology and equipment and offering trial hearing aids.

#### Step 4: Hearing Aid Trial and Fitting—Face-to-Face

Patients agreed and strongly agreed that a hearing aid trial helped to experience the difference that hearing aids can make in their life (26/27, 96%). All patients who acquired their hearing aids (steps 4-5) agreed and strongly agreed that the hearing aid trial and its usage was beneficial (Table 4).

**Table 4:** Patient evaluation of the hearing aid trial and fitting.

Questions related to the hearing aid trial and fitting	Strongly disagree, n (%)	Disagree, n (%)	Neutral, n (%)	Agree, n (%)	Strongly agree, n (%)
The hearing aid trial helped me experience the difference hearing aids can make in my life (N=27)	0 (0)	0 (0)	1 (4)	5 (19)	21 (78)
The opportunity to try hearing aids helped me make an informed decision to buy hearing aids (N=13)	0 (0)	0 (0)	0 (0)	5 (39)	8 (62)
I felt it was easy to use the hearing aids in the trial period which gave me the confidence in my ability to use it on my own (N=13)	0 (0)	0 (0)	0 (0)	5 (39)	8 (62)
I trust that the hearing aids will assist me to hear better in my daily life (N=13)	0 (0)	0 (0)	0 (0)	4 (31)	9 (69)
The time I had to trial the hearing aids in my daily life (home/work) was adequate (N=13)	0 (0)	0 (0)	0 (0)	3 (23)	10 (77)
My quality of life has improved by using my hearing aids (N=13)	0 (0)	0 (0)	0 (0)	4 (31)	9 (69)

Of those patients who were fitted with hearing aids (steps 4-5), the majority (6/9, 67%) complimented the service received, were satisfied with the care offered, and did not have suggestions for service improvements. Reasons for patients not continuing with HHC (11/18, 61%) included cost as a prohibitive factor (7/18, 39%), concerns regarding the stigma of wearing hearing aids (3/18, 17%), and belief that the hearing loss was not severe enough to warrant the use of hearing aids (3/18, 17%). One person suggested a financing option to make hearing aids more affordable.

#### Step 5: Audiological Rehabilitation—Web-Based and Face-to-Face

Except for 1 person, all patients agreed and strongly agreed that the online audiological rehabilitation was helpful (8/9, 89%). In addition to the program, support was offered to patients as required both online and by face-to-face methods.

### Overall Satisfaction—Web-Based and Face-to-Face Clinic Services

The mean SAPS score of the 31 patients reported was 26 (SD 3; Table 5). There were only 3 instances where 1 patient was unsure (neither satisfied nor dissatisfied) regarding his or her satisfaction in terms of the effect of the HHC treatment, choices available to the patient, and dissatisfaction with the care received. In total, 3 patients (3/31, 10%) felt that the time with the clinic audiologist was too short.

The NPS score was 87, which indicates that patients are highly likely to recommend the clinic to friends and family. The majority of patients (21/31, 68%) provided reasons for their rating including competence, result-driven exceptional service (11/31, 35%), tailored service (4/31, 13%), and reliable and efficient service (2/31, 6%).

The three most important reasons for continuing with HHC services with the hybrid clinic were as follows: personalized care and understanding audiologist, who is patient and accommodating (11/31, 36%); confidence in the audiologist, kind, caring, helpful, caring, efficient (8/31, 26%); and technical knowledge of the product and equipment (5/31, 17%).

A significant positive correlation was found between age and the number of appointments (*r*=0.367; *P*=.007) and a positive but not significant correlation (*r*=0.216; *P*=.12) was reported between age and the number of support instances.

**Table 5:** Overall Short Assessment of Patient Satisfaction scores categorized according to “Very dissatisfied,” “Dissatisfied,” “Satisfied,” and “Very Satisfied” for patients who sought hearing health care (N=31).

SAPS^a^ category	Range of score	Frequency (%)
Very dissatisfied	0-10	0 (0)
Dissatisfied	11-18	1 (3)
Satisfied	19-26	14 (45)
Very satisfied	27-28	16 (52)

^a^SAPS: Short Assessment of Patient Satisfaction.

## Discussion

### Hybrid Hearing Health Care Delivery Model

This study provides insights into a hybrid service delivery model that assessed adult patients’ perspectives on online and face-to-face services offered. An asynchronous Web-based hearing screening successfully recruited patients seeking HHC online. Patient experiences with this online screening test were positive and, together with motivational engagement, were rated as time-efficient, valuable, and supporting continuation with HHC. This study employed nonopportunistic testing as participants actively opted to visit the website and complete the online hearing screening test. The potential reasons for mixed findings on the ease of testing, which comprised 23 user entries, could be due to the internet speeds (Wi-Fi or mobile data 3G or 4G) in terms of wait time when loading the widget on mobile devices or computers, proficiency with the digital device, or the actual test duration. Hearing screening tests are typically offered in isolation, and longitudinal studies show that a significant percentage of people do not follow-up with diagnostic measures and rehabilitation [47-49]. Approximately 75% (38/51) of patients who failed an online hearing screening test did not continue with HHC as reported in our previous study [27]. Another study reported that older adults who were considering or preparing to take action for their hearing loss were willing to access online HHC and that a simple user interface and short-term training may optimize the usability of online HHC programs for them [50]. In line with this, this study offered hybrid diagnostic and rehabilitative HHC services directly following the hearing screening. This is the first report to perform a process evaluation of a hybrid model of HHC. Previous reports focused on the validation of these tools and not on patient experiences [47,51].

WhatsApp messaging was rated highly, and patients were satisfied with this mode of communication. Patients used and preferred WhatsApp messaging as the primary communication method with the clinic where a dedicated mobile phone with WhatsApp, phone calls, and email was set up for this hybrid clinic. In other health professions, physicians have successfully incorporated WhatsApp into clinical practice with no need for further training or technical competency building [52].

The advantages and disadvantages of using WhatsApp in clinical practice are well documented within health care [53]. However, there is no uniformity in the usage of WhatsApp, as a recent study reports that doctors were more likely to use WhatsApp in patient communication or share information with colleagues than nurses [54]. Research evidence suggests that WhatsApp can be a promising tool that allows health professionals and the general public to communicate or allows communication among health care professionals themselves to compare and learn from each other [55]. There is still a need for high-quality research to evaluate the value and risks of using it as a health communication tool [54,55].

The diagnostic hearing evaluation (step 3) was an integral step to establish a therapeutic relationship in this hybrid model. A strength of this model was that the therapeutic relationship already commenced before the face-to-face appointment (step 3) and was continued through the patient’s HHC journey with the same clinic audiologist either online or in-person. The benefit of clinician continuity is mixed; in a physician environment, seeing a known provider is found to be beneficial in terms of a cost-benefit factor [56], whereas in an audiological setting, no difference was noted on hearing aid outcomes when patients are attended to by different clinicians [57].

Previous tele-audiology studies have taken steps toward investigating patient satisfaction within remote hearing aid fittings [21], services [22], and programming and fitting [58] with reasonable patient satisfaction noted. However, the first 2 studies reported findings based on standardized hearing aid outcome measures (International Outcome Inventory for Hearing Aids and Satisfaction with Amplification in Daily Living) rather than a process evaluation of patient experiences and satisfaction with HHC [17,18]. The last study [58] measured patient experience satisfaction using a validated questionnaire and found that patient satisfaction with hearing aid programming and fitting via tele-audiology versus face-to-face was the same.

The online audiological rehabilitation offering was reported as a positive addition to this hybrid clinic’s services. eHealth might be a viable option to offer tele-audiology services to both adult patients and their significant other as they already use internet-connected technologies to access health care, and this could promote patient-centered care from a biopsychosocial context [59]. Telehealth interventions for audiology are expanding, and research conducted on audiological, vestibular, and tinnitus rehabilitation show promising results [25].

### Overall Satisfaction

Patient satisfaction in this study, which used 5 steps in a hybrid HHC service delivery model, was found to be higher than previously published SAPS data. In this study, the SAPS mean score was 26 (SD 3) as compared with findings from an incontinence clinic (mean SAPS score 22, SD 5) [38] and a psychiatry clinic (mean SAPS score 8, SD 4) [42]. The NPS score in this study was high (87) in comparison with an NPC score of 52 in a study of 728 patients who rated their satisfaction with synchronous videos across the health department [60]. NPS scores from another health field in the National Health System in the United Kingdom reported the following scores, however the response scale was slightly altered from the original: joint replacement was 60 with individual scores for total hip replacement and total knee replacement of 71 and 49, respectively [61]. Other researchers have highlighted the attractiveness of adapting the NPS for health care as it is less reliant on the literacy of responders, limited resources are needed to adapt the tool, and it provides more valuable information than a binary yes or no scale [62].

The audiologist’s clinical engagement and professional services were identified as essential components in the positive patient experiences in this study. Previous research also indicates that patients prefer patient-centered interactions with a health professional, and this is associated with high satisfaction [63]. Offering patient-centered care has also been proposed as a way to improve hearing aid adoption [64].

Even though 61% (19/31) of patients experienced previous HHC services from other audiologists or clinics, 95% (18/19) rated the services offered in this hybrid clinic more favorably. Patient experience and satisfaction were equally high and positive in both online and face-to-face service offerings in this hybrid clinic. However, there is still a paucity of evidence regarding the uptake of eHealth HHC, its effectiveness, and the satisfaction of patients using such service delivery models. As technology evolves, so will the continuum of direct-to-consumer and traditional face-to-face models. This study applied online and face-to-face components into a hybrid clinic and measured high patient satisfaction through a process evaluation. This model still required the need for 1 or 2 face-to-face appointments with the audiologist compared with more traditional clinical pathways. The fact that older patients needed more appointments may indicate that more audiological support is needed in the initial stages of adapting to hearing aids where additional support could be offered using asynchronous methods. This study provides initial evidence that can support audiologists who are limited in numbers but are required to provide services to a large area. This model may also provide patients with an alternative service delivery model, who could benefit from a combination of online and face-to-face appointments. Individual audiologists can customize this hybrid model to meet the needs of their patient demographic and for those patients willing to seek HHC differently.

This study offered individuals searching for HHC within the target location with an online hearing screening test as the first action to initiate care. Combining online and face-to-face communication methods also allowed patients to stay in touch with the audiologist when needed. Patients paid for their HHC services, removing volunteer biases and highlighting the potential of this model to translate into a scalable clinical practice. However, patients who pay for their hearing aids could introduce another bias or view services as more favorable, and this could be considered a limitation. Another limitation of the study is the lack of a comparator to establish whether this hybrid model was better or worse as compared with more traditional HHC delivery models where satisfaction could be measured as being similar in face-to-face only with no online services employed. The fact that the same person served as the clinician and as the researcher collecting the online questionnaires and that patients could have been influenced to provide favorable ratings (social desirability bias) could also be considered limitations in this study. The completion of the questionnaire is also vulnerable to both nonresponse bias (15/46, 33% of patients did not respond to the questionnaire) and recall bias. It is not possible to separate the influence of the audiologist’s skills versus the hybrid model when analyzing the patients’ satisfaction with the care received. This study also had a relatively small sample within a defined area of South Africa that required patients to have internet access and the necessary digital skills to complete an online hearing screening test, which limits generalizability. Future studies in modifications to the service delivery models would benefit from a comparator group designed into research studies and to test mobile and computer proficiency and the effects of age on the uptake of HHC in such a hybrid model. Another future consideration would be to document the long-term effects in terms of economic viability and scalability of such a model. This hybrid model is the first concept to be tested, and we foresee modifications to this service delivery model made possible in the future when technology advances to facilitate more audiological services remotely to meet the needs of the patient and the audiologist.

### Conclusions

In conclusion, the positive patient experience and satisfaction demonstrates the potential of hybrid online and face-to-face HHC to meet patient needs. Sustainable and scalable service delivery models that incorporate eHealth are required to meet the challenges of untreated hearing loss globally.

## Appendix

Multimedia Appendix 1 Online questionnaire. Patient experience and satisfaction with hearing health care received.

Multimedia Appendix 2 Framework for the process evaluation of Web-based and face-to-face services offered in the hybrid hearing health care model.

## Abbreviations

eHealth: electronic health
HHC: hearing health care
NPS: net promoter score
SAPS: Short Assessment of Patient Satisfaction
SNR: signal-to-noise ratio
