# Supplementary material for: Patient Uptake, Experience, and Satisfaction Using Web-Based and Face-to-Face Hearing Health Services: Process Evaluation Study
Source: J Med Internet Res. 2020 Mar 20;22(3):e15875. doi: 10.2196/15875 (PMC7125439; doi:10.2196/15875)
Supplement: Multimedia Appendix 1 [file jmir_v22i3e15875_app1.docx]

## Supplementary file:

## Appendix 1: Online questionnaire

## Patient experience and satisfaction with hearing health care received

| **Section A: Short Assessment of Patient Satisfaction [as per reference 40]** |
| --- |

| **Section B: Process Evaluation**  Please answer the questions on the following pages relating to the steps you have completed in seeking help with the Hearing Research Clinic NPC. Click on the arrow to continue. |
| --- |
| 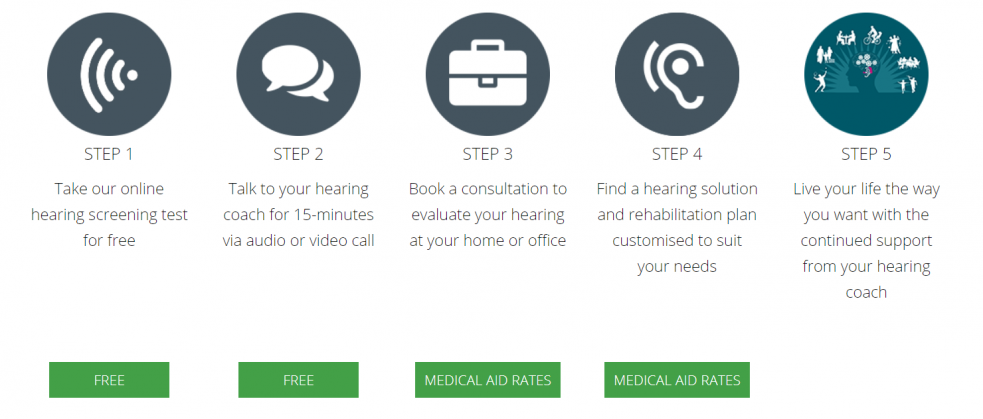 |
| 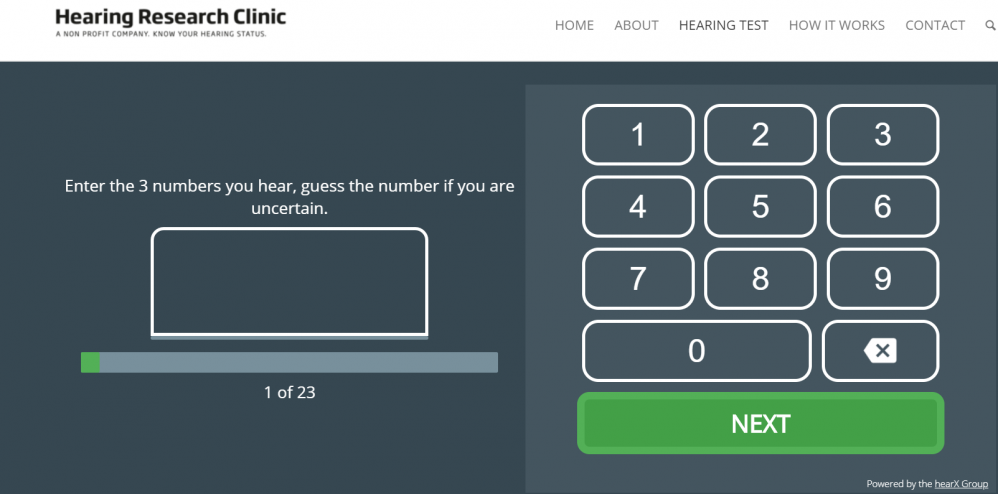**Step 1: Online hearing screening test**    Do you remember taking an online hearing screening test, which played numbers in background noise? If ‘no’, select "I did not complete this step (N/A)", if ‘yes’, go ahead as is. Click on the arrow to continue. |
| Please answer these questions related to the online hearing screening test:   \|  \| Strongly disagree (1) \| Disagree (2) \| Neutral (3) \| Agree (4) \| Strongly agree (5) \| I did not complete this step (N/A) (6) \| \| --- \| --- \| --- \| --- \| --- \| --- \| --- \| \| B1. Taking the online test was simple (1) \|  \|  \|  \|  \|  \|  \| \| B2. Taking the online test was quick (2) \|  \|  \|  \|  \|  \|  \| \| B3. Taking the online test was informative (3) \|  \|  \|  \|  \|  \|  \| \| B4. I found this online test easy to use (4) \|  \|  \|  \|  \|  \|  \| \| B5. I thought the online test was fast (5) \|  \|  \|  \|  \|  \|  \| \| B6. The test result seemed reliable (6) \|  \|  \|  \|  \|  \|  \| \| B7. Taking this online test has helped me to take the next steps to improve my hearing (7) \|  \|  \|  \|  \|  \|  \| |
| 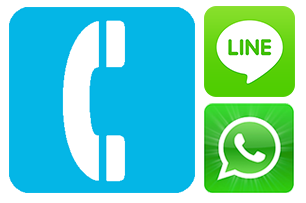**Step 2: Phone call/WhatsApp**  Do you remember talking to us on the phone or by WhatsApp to discuss your hearing challenges?’ If ‘no’, select "I did not complete this step (N/A)", if ‘yes’, go ahead as is. |
| Please answer these questions related to the phone call/WhatsApp messaging to assess your hearing challenges:   \|  \| Strongly disagree (1) \| Disagree (2) \| Neutral (3) \| Agree (4) \| Strongly agree (5) \| I did not complete this step (N/A) (6) \| \| --- \| --- \| --- \| --- \| --- \| --- \| --- \| \| B8. The phone call/WhatsApp was informative (1) \|  \|  \|  \|  \|  \|  \| \| B9. The phone call/WhatsApp was an easy way for me to communicate with the audiologist/clinic (2) \|  \|  \|  \|  \|  \|  \| \| B10. The phone call/WhatsApp helped me in taking the next step (3) \|  \|  \|  \|  \|  \|  \| \| B11. The phone call/WhatsApp provided me with relevant information regarding my hearing (4) \|  \|  \|  \|  \|  \|  \| \| B12. The phone call/WhatsApp helped me to take the next step and book my hearing evaluation consulation (5) \|  \|  \|  \|  \|  \|  \| \| B13. The phone call/WhatsApp was a quick way for me to communicate with the audiologist/clinic (6) \|  \|  \|  \|  \|  \|  \|   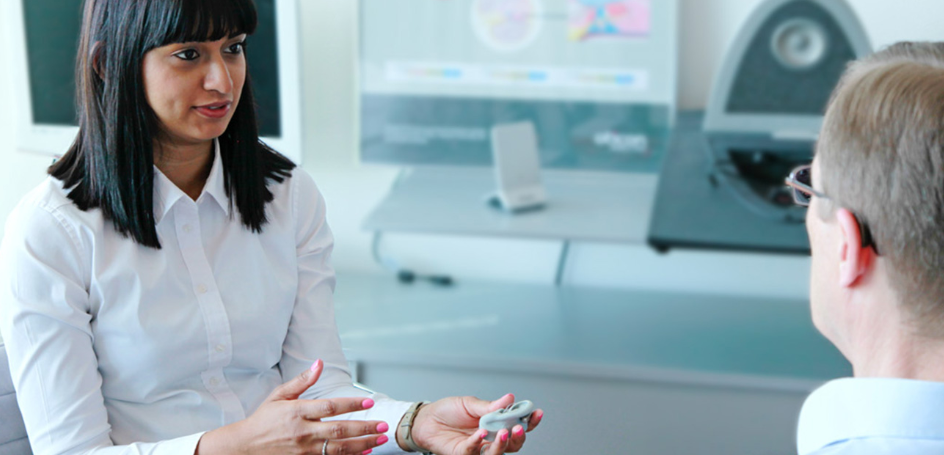**Step 3: Hearing evaluation consultation**  Do you remember completing your hearing evaluation with our Audiologist? If ‘no’, select "I did not complete this step (N/A)", if ‘yes’, go ahead as is.  Click on the arrow to continue. |
| Please answer these questions related to the diagnostic hearing test you completed with our audiologist:   \|  \| Strongly disagree (1) \| Disagree (2) \| Neutral (3) \| Agree (4) \| Strongly agree (5) \| I did not complete this step (N/A) (6) \| \| --- \| --- \| --- \| --- \| --- \| --- \| --- \| \| B14. The diagnostic hearing test was comprehensive (1) \|  \|  \|  \|  \|  \|  \| \| B15. The audiological consultation provided me with the information I needed (2) \|  \|  \|  \|  \|  \|  \| \| B16. The diagnostic hearing test was an easy test to complete with the guidance from the audiologist (3) \|  \|  \|  \|  \|  \|  \| \| B17. It was beneficial to have a hearing aid trial option available after my diagnostic hearing test (in the first consultation) (4) \|  \|  \|  \|  \|  \|  \| \| B18. It was easy to use the hearing aid during the trial period offered to me (5) \|  \|  \|  \|  \|  \|  \| \| B19. I trust the results from my diagnostic hearing test (6) \|  \|  \|  \|  \|  \|  \| \| B20. The time spent on my diagnostic hearing test was adequate (7) \|  \|  \|  \|  \|  \|  \| |
| **Step 4: Your customised hearing solution and rehabilitation plan**  Do you remember being fitted with your trial hearing aids or to purchase your own hearing aids? If ‘no’, select "I did not complete this step (N/A)", if ‘yes’, go ahead as is.  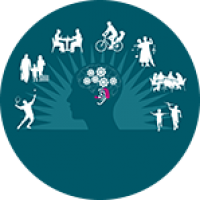 |
| Please answer these questions related to your hearing aid trial:   \|  \| Strongly disagree (1) \| Disagree (2) \| Neutral (3) \| Agree (4) \| Strongly agree (5) \| I did not complete this step (N/A) (6) \| \| --- \| --- \| --- \| --- \| --- \| --- \| --- \| \| B21. The hearing aid trial helped me experience the difference hearing aids can make in my life (1) \|  \|  \|  \|  \|  \|  \| |
| **The following questions B22-B25 -only shown to group who discontinued HHC (did not complete all five steps):**  B22. Reasons why, I did not proceed with purchasing my hearing aids (select all that apply):  (*Randomization of answers was implemented by online software)*   - I believe hearing aids are too costly (1) - I am concerned about the way hearing aids would look on me (2) - I am concerned about what my family and friends would think of me as a person wearing hearing aids (3) - I do not believe I could use hearing aids (4) - I do not believe my hearing impairment is bad enough yet to seek hearing health care (5) - I did not try any hearing aids (7) - Other, please explain: (6) ________________________________________________ |
| B23. What would you need to continue with treatment for your hearing loss?  ________________________________________________________________  ________________________________________________________________ |
| B24. Have you gone elsewhere to get help for your hearing challenges?  Yes (4)  No (5) |
| Display This Question:  If B24. Have you gone elsewhere to get help for your hearing challenges? = Yes  Or B24. Have you gone elsewhere to get help for your hearing challenges? = No  B25. Please explain why?  ________________________________________________________________ |
| **The following questions B22B-B27B -only shown to group who continued with HHC (completed all five steps):**   \|  \| Strongly disagree (1) \| Disagree (2) \| Neutral (3) \| Agree (4) \| Strongly agree (5) \| I did not complete this step (N/A) (6) \| \| --- \| --- \| --- \| --- \| --- \| --- \| --- \| \| B22B. The opportunity to try hearing aids helped me make an informed decision to buy hearing aids (2) \|  \|  \|  \|  \|  \|  \| \| B23B. I felt it was easy to use the hearing aids in the trial period which gave me the confidence in my ability to use it on my own (3) \|  \|  \|  \|  \|  \|  \| \| B24B. I trust that the hearing aids will assist me to hear better in my daily life (4) \|  \|  \|  \|  \|  \|  \| \| B25B. The time I had to trial the hearing aids in my daily life (home/work) was adequate (5) \|  \|  \|  \|  \|  \|  \| \| B26B. My quality of life has improved by using my hearing aids (6) \|  \|  \|  \|  \|  \|  \| \| B27B. The online programme was helpful to me (7) \|  \|  \|  \|  \|  \|  \| |
| **Section C: Personal preferences**  C1. Select all the types of communication that you **used** to stay in touch with your audiologist:   - Phone call (1) - Email (2) - WhatsApp (3) - SMS messages (4) - Facebook (5) - Other, please specify (6) ________________________________________________ |
| C2. Select all the types of communication that you **prefer** to use in order to stay in touch with your audiologist:   - Phone call (1) - Email (2) - WhatsApp (3) - SMS messages (4) - Facebook (5) - Other, please specify (6) ________________________________________________ |
| C3. Think about **how much you used** your present hearing aid(s) over the past two weeks (during your trial period). On an average day, how many hours did you use the hearing aid(s)?  None (1)  Less than 1 hour a day (2)  1 to 4 hours a day (3)  4 to 8 hours a day (4)  more than 8 hours a day (5) |
| C4. Have you had **previous** hearing tests/evaluations completed by another clinic/audiologist?  Yes (1)  No (2) |
| Display This Question:  If C4. Have you had previous hearing tests/evaluations completed by another clinic/audiologist? = Yes  C5. How do you **rate** **your experience** with this clinic (Hearing Research Clinic NPC) compared to your past experiences?  Worse (1)  Same (2)  Better (3) |
| Display This Question:  If C4. Have you had previous hearing tests/evaluations completed by another clinic/audiologist? = Yes  C6. What's different **compared** to your previous experiences with other audiologists?  ________________________________________________________________  ________________________________________________________________ |
| C7. What made you **continue** with your treatment plan with the Hearing Research Clinic NPC? Name your top 3 reasons.  ________________________________________________________________  ________________________________________________________________  ________________________________________________________________ |
| C8. On a scale from 0-10, how **likely are you to recommend** this clinic (Hearing Research Clinic NPC) to your friends and family?  0 (0)  1 (1)  2 (2)  3 (3)  4 (4)  5 (5)  6 (6)  7 (7)  8 (8)  9 (9)  10 (10)  Please explain your rating above:  ________________________________________________________________  ________________________________________________________________ |
| C9. How do you think we can **improve our services**?  ________________________________________________________________  ________________________________________________________________  ________________________________________________________________  ________________________________________________________________ |
| **END** |
