# Supplementary material for: Patient Uptake, Experience, and Satisfaction Using Web-Based and Face-to-Face Hearing Health Services: Process Evaluation Study
Source: J Med Internet Res. 2020 Mar 20;22(3):e15875. doi: 10.2196/15875 (PMC7125439; doi:10.2196/15875)
Supplement: Multimedia Appendix 2 [file jmir_v22i3e15875_app2.docx]

Multimedia Appendix 2. Framework for the process evaluation of Web-based and face-to-face services offered in the hybrid hearing health care model

| Process evaluation categories as inspired by Linnan and Steckler [38] | Study process that supports this model | Persons involved |
| --- | --- | --- |
| Clarify the theory that underlies intervention development | Literature study search:   - General eHealth^a^ - eHealth in hearing care - Motivation/readiness engagement within health psychology and behavioral sciences - Patient satisfaction in health care | - Primary investigator (first author) - Collaborators (second and third authors) - Industry experts |
| Create theory-informed interventions | - A patient satisfaction survey found (SAPS^b^) [40] - Process evaluation [38] - Qualitative, open-ended question formulation within a health care service delivery model - NPS^c^ [43] | - Primary investigator (first author) - Collaborators (second and third authors) - Industry experts |
| Create an inventory of process objectives | - Alignment with key stakeholders on the study objectives and the knowledge generation of hybrid HHC^d^ services and learning about patient behaviors and preferences | - Primary investigator (first author) - Collaborators (second and third authors) - Industry experts |
| Reach consensus on the process evaluation questions to be answered | - Pilot conducted on a process evaluation questionnaire with 10 independent researchers within the HHC field - Consultation and alignment with a statistician regarding the internal validity and robustness of the questionnaire - Alignment with key stakeholders of the final themes and questions included into the questionnaire | - Primary investigator (first author) - Collaborators (second and third authors) - Independent researchers - Statistician |
| Identify or create measurement tools to assess prioritized process objectives | - The first section in the online questionnaire incorporated a validated measure of general patient satisfaction (SAPS) - The second section incorporated all 5 steps, allowing for patient feedback on each step and a component they encountered of the hybrid model - The third section included open-ended questions, as well as a comparison with previous care and a rating of the services received using the net promoter score | - Primary investigator (first author) - Collaborators (second and third authors) - Industry experts |
| Design, implement, and administer quality control assurances | - Design: The design was based on the university-accepted online questionnaire design - Implement: Online questionnaires were sent using patients’ email addresses and were then coded to ensure anonymity before analysis commenced - Quality control and assurances: The questionnaire was tested by 5 independent researchers using the online software, Qualtrics, for correct transitioning, flow, quality, and compatibility with different devices - Data were downloaded and stored offline every month by the first author | - Primary investigator (first author) - Collaborators (second and third authors) - Independent researchers |
| Collect, manage, and clean data | - Data were collected over a period of 3 months - Data were only accessed by the clinic audiologist - Data were coded to ensure anonymity - Data were cleaned and coded before the analysis began | - Primary investigator (first author) - Participants in this study |
| Analyze data | - Coded data were sent to a statistician to assist with data analysis | - Primary investigator (first author) - Statistician |
| Create user-friendly reports on the selected process objectives | - Dissemination of results by conference presentations (poster and podium) - Results shared in a peer-reviewed ISI-accredited journal | - Primary investigator (first author) - Collaborators (second and third authors) - Industry experts |
| Refine theory, interventions, measurements, and analysis tools | - On the basis of the results from the 5 steps of the patient journey included in this process evaluation, strengths and limitations were highlighted - The suggested recommendations are outlined - Future research considerations are outlined | - Primary investigator (first author) - Collaborators (second and third authors) |

^a^eHealth: electronic health.

^b^SAPS: Short Assessment of Patient Satisfaction.

^c^NPS: net promoter score.

^d^HHC: hearing health care.
